# Antiviral Treatment for Hepatitis C Virus Infection after Liver Transplantation

**DOI:** 10.1155/2010/475746

**Published:** 2010-11-01

**Authors:** Yasuhiko Sugawara, Sumihito Tamura, Norihiro Kokudo

**Affiliations:** Artificial Organ and Transplantation Division, Department of Surgery, Graduate School of Medicine, University of Tokyo, 7-3-1 Hongo, Bunkyo-ku, Tokyo 113-8655, Japan

## Abstract

A significant proportion of patients with chronic hepatitis C virus (HCV) infection develop liver cirrhosis and complications of end-stage liver disease over two to three decades and require liver transplantation, however, reinfection is common and leads to further adverse events under immunosuppression. Pretransplant antiviral or preemptive therapy is limited to mildly decompensated patients due to poor tolerance. The mainstay of management represents directed antiviral therapy after evidence of recurrence of chronic hepatitis C. Combined pegylated interferon and ribavirin therapy is the current standard treatment with sustained viral response rates of 25% to 45%. The rate is lower than that in the immunocompetent population, partly due to the high prevalence of intolerability. To date, there is no general consensus regarding the antiviral treatment modality, timing, or dosing for HCV in patients with advanced liver disease and after liver transplantation. New anti-HCV drugs to delay disease progression or to enhance viral clearance are necessary.

## 1. Introduction

According to the World Health Organization, 130 to 170 million people are chronically infected with hepatitis C virus (HCV) and 3 to 4 million people are newly infected each year worldwide [1]. The median time to develop cirrhosis is estimated to be 30 years, and 33% of patients have an expected median time to the development of cirrhosis of less than 20 years. Although antiviral therapy is available, the number of patients with end-stage liver disease due to HCV will continue rise over the next 10 years [2]. HCV is the major cause of chronic liver disease, cirrhosis, and liver cancer in most developed countries [3], including Japan [4]. It is the most common indication for liver transplantation in developed nations [5, 6]. 

Liver transplantation is an effective treatment to reduce morbidity and mortality in this population. Reinfection with HCV, however, is a critical complication with major effects on graft and patient survival. Indeed, the real challenge of controlling HCV begins after liver transplantation under life-long immunosuppression, and mitigating the damage is a crucial concern. In this paper, we focus on this challenging aspect, evaluating the available treatment options and strategies against HCV before and after transplantation to prevent reinfection with the goal of eradicating recurrent infection.

## 2. Clinical Course after Liver Transplantation

Spontaneous clearance of HCV after transplantation is rare [7–11], and reinfection of the allograft is common [12, 13]. Histologic progression of HCV under immunosuppressive therapy is more rapid than that in nontransplant patients [14, 15]. HCV patients have a poorer prognosis after liver transplantation than those with other indications [16–19], a finding that remains unchanged in recent research [20]. Large studies have demonstrated that recipients with HCV have approximately 10% lower 5-year graft and patient survival rates than non-HCV controls [18, 19]. One study comparing approximately 7500 HCV recipients and 20,000 non-HCV recipients reported an overall 3-year patient survival rate of 79% in HCV patients and 81% in non-HCV patients [16]. Factors with a significant negative impact on patient survival include a preoperative model for end-stage liver disease (MELD) score [21], fibrosis stage of 2 or greater at 12-month biopsy, advanced donor age, history of hepatocellular carcinoma (HCC), and early acute rejection [22].

HCV reinfection occurs during transplantation in the reperfusion phase of the graft, and acute hepatitis can usually be detected 1 to 3 months after transplantation [23]. The clinical course following reinfection varies. In general, 8% to 30% of the recipients will present with severe progressive disease within 5 years [15, 17, 24]. The median time to cirrhosis in the nontransplant patients was 30 years [25] and in the transplanted patients with HCV disease is expected to be 10 years. The risk of decompensation is 50% within 1 year after diagnosis in the absence of therapeutic intervention [15, 26]. A small percentage of recipients can develop an early cholestatic hepatitis within the first year after transplantation with a risk (2%–8%) of progressive liver dysfunction and rapid development of cirrhosis [12, 27]. Retransplantation in these patients is associated with poor outcomes and is controversial [28].

## 3. Risk Factors for Severe Recurrent HCV

The reported risk factors include advanced donor age [29, 30], high viral load in the preoperative or early postoperative periods [31, 32], treatment of acute rejection [28], long duration between the antiviral therapy and transplantation [16, 18, 33], and baseline pre transplant liver function [34]. The expression of cytokeratin 19 and that of vimentin in liver biopsies without fibrotic changes (F0) [35] are also considered risk factors for severe recurrent HCV. Postoperative insulin resistance diabetes mellitus [36, 37], metabolic syndrome [38], and lipid peroxidation (oxidative degradation of lipids) [39] are reported to be associated with severe HCV recurrence. 

The role of corticosteroids in severe recurrent HCV is complicated. Steroid bolus injection as acute rejection therapy is one of the risk factors for severe HCV recurrence and graft loss [19]. Rapid withdrawal of corticosteroids might cause graft fibrosis [40], but a corticoid-free regimen may be promising [41]. A meta-analysis of randomized trials using a corticoid-free immunosuppression regimen showed a significant reduction of HCV recurrence in corticoid-free groups [42]. 

The impact of other immunosuppressive agents, including mycophenolate mofetil, azathioprine [43], or interleukin-2 inhibitors [44] on severe recurrent HCV remains controversial. OKT3 is associated with increased graft loss and increased mortality [12].

A viral quasispecies is a group of viruses related by a similar mutation or mutations, competing within a highly mutagenic environment. After transplantation, diversification of hypervariable region 1 is decreased, and the virus population becomes more homologous [45] although the changes might be temporary [46]. A more complex HCV hypervariable region 1 quasispecies population was reported to be associated with HCV recurrence after transplantation [47, 48].

## 4. Viral Kinetics

Powers et al. [49] confirmed that viral loads begin to rise 15 hours after the anhepatic phase. In total, 19% hepatocytes are infected in an average of 37 days (range: 4–82 days) after transplantation. Schiano et al. [50] demonstrated accelerated HCV kinetics in LDLT recipients (*n* = 11) compared to deceased donor liver transplantation (DDLT) recipients (*n* = 15). In their study, HCV RNA levels rose more rapidly in LDLT patients; the differences in patient and graft survival, however, did not reach statistical significance. 

Another study [51] focusing on the histologic aspects of HCV recurrence with protocol biopsy reported more severe progression of HCV disease in LDLT compared to DDLT. Other studies [52, 53] have also demonstrated that individuals with a high level of replication in the perioperative period develop more fibrosis in the allograft 1 year after transplantation. When the graft develops fibrotic or cirrhotic changes, the patient prognosis is dismal.

## 5. Pretransplant Antiviral Therapy

Antiviral treatment of patients with mildly decompensated cirrhosis (model for end-stage liver disease, MELD >18) is a more problematic approach than treatment after transplantation.

Suitable candidates for pretransplant therapy would be treatment-naïve patients or prior relapsers to standard interferon (IFN) and ribavirin (RBV) treatment because the chances for an on-treatment virologic response before transplantation are high [27]. Patients with predictable timing of transplantation such as those with living donors or those with HCC may be good candidates for pre-transplant therapy. The use of growth factors is considered an option to treat therapy-associated anemia and leucopenia as it can improve quality of life and may decrease the need for antiviral therapy dose reduction [54].

Everson et al. [55] reported his experience in the treatment of 102 patients with chronic hepatitis and decompensated liver disease using a low accelerating dosage regimen of IFN alfa-2b plus RBV. Serum HCV RNA was cleared in approximately 40% of patients on this treatment, and 22% achieved a sustained virologic response (SVR). 

Forns et al. [56] administered antiviral therapy to patients with IFN alpha-2b 3 MU/day and RBV 800 mg/day when the expected time until transplantation was less than 4 months. Of 30 patients enrolled, 9 (30%) achieved a virologic response and 21 did not respond to therapy. Of the nine, six remained free of infection after a median followup of 46 weeks and HCV infection recurred in three patients after transplantation. In contrast, Smallwood et al. [57] reported that patients treated with IFN before transplantation have a significantly earlier and more aggressive recurrence of HCV. Carrión et al. [58] performed a case control study comparing 51 patients who underwent treatment with IFN and RBV and a control group (untreated 51 individuals awaiting transplantation who were matched by age, Child-Pugh, and time on the waiting list). There was a higher incidence of bacterial infections after transplantation in treated patients, particularly in Child-Pugh B-C individuals. Further data are needed before a definitive conclusion can be drawn.

## 6. Early Posttransplant Antiviral (Preemptive) Therapy

### 6.1. Its Rationale and Use in LDLT Patients

Preemptive treatment may be beneficial in potentially tolerant patients, such as patients with a lower natural MELD score undergoing liver transplantation for HCC within the Milan criteria, with an outcome comparable to that of non-HCC cases, and also in well-planned LDLT cases [59] with splenectomy [60]. The preemptive approach may provide an oncologic benefit, which has been demonstrated in nontransplant patients with HCV who underwent liver resection for HCC [61]. 

The rationale for preemptive therapy is to strike at a time when the total HCV viral load is relatively low after liver transplantation [23, 49] and histologic damage is absent or minimal. Delay of histologic damage can be expected [62, 63]. Garcia-Retortillo et al. [23] observed a rapid decrease in the viral load during the anhepatic phase in 20 patients who underwent liver transplantation for HCV. The viral load continued to exponentially decrease after graft reperfusion with a progressive increase after the first week of transplantation, reaching a plateau by the end of the first month. 

We examined the feasibility and efficacy of a preemptive combination of IFN and RBV therapy against HCV in LDLT patients [64]. Tolerance was a limiting factor, with 25% of the patients deviating from the planned protocol. Overall, among the 23 patients enrolled, 9 (39%) achieved SVR. The cumulative 3-year survival rate did not differ between the enrolled patients and the HCV-negative patients (90%) during the study period [59]. The results of the study were encouraging and demonstrated an optimal window for treatment initiation and the optimal doses of IFN and RBV necessary for effective viral eradication [65] after LDLT.

### 6.2. In DDLT

The overall efficacy and feasibility of preemptive therapy in DDLT is controversial. Randomized studies on a preemptive approach with IFN monotherapy [66, 67] or combined therapy [63] demonstrated a reduced or delayed incidence of hepatitis after liver transplantation, but not the prevention of viremia. Discontinuation of the treatment was necessary in 30% of the subjects in the study. Mazzaferro et al. [68] treated 36 recipients with IFN-alpha-2b (3 million IU, 3 times/week) and RBV (10 mg/kg per day). The treatment was initiated at a median of 18 days after the operation and continued for a year. After a median followup of 52 months, 5-year patient survival was 88%. Serum HCV RNA was cleared in 12 patients of the 36 recipients (33%) after a median of 37 days. These patients remained negative for serum HCV RNA for a median of 36 additional months without antiviral treatment. Dose reduction was necessary in 9 (25%) patients. Although these outcomes were encouraging, the current understanding is that the adverse events associated with treatment outweigh the theoretical benefits of preemptive therapy [12]. 

Shergill et al. [69] reported about the results of preemptive treatment. Only 51 (41%) of 124 transplant recipients were eligible for preemptive treatment; eligible patients had lower MELD and Child-Pugh scores pretransplantation. Dose reductions and discontinuations were required in 85% and 37% of patients, respectively, and 27% experienced serious adverse events. Only 15% of patients were able to achieve full-dose treatment during treatment. End-of-treatment rate and SVR were 14% and 9%, respectively.

## 7. Treatment for Established Infection

The recommendation of the International Liver Transplantation Society is to perform post-transplant surveillance and protocol biopsies to detect recurrent HCV disease [70] and to start combined pegylated-(peg-) IFN and RBV therapy for stage II fibrosis [12]. Treatment of HCV recurrence is mostly modeled after the strategy of treating HCV in nontransplant patients [5, 6]: Studies of a noncontrolled series of patients [71–81] revealed an efficacy of 26% to 50% after peg-IFN and RBV therapy, a higher efficacy than that of conventional IFN and RBV therapy. 

These studies suggested that factors associated with higher probabilities of a viral response were nongenotype 1, low pretreatment HCV RNA levels, absence of advanced cirrhosis, early virologic response (≥2 log drop in HCV RNA from baseline at 3 months), and adherence to combination therapy [71, 82–93] similar to that in nontransplant patients. Other reports [90, 94] suggested that patients on cyclosporine had a higher SVR. Berenguer et al. [73] reported a higher SVR rate for nongenotype 1 (60%) versus genotype 1 (31%) and a higher SVR rate with peg-IFN with RBV compared to standard IFN with RBV (50% versus 13%). Combined treatment with peg-IFN is suggested to be effective among those who failed previous conventional IFN and RBV therapy, achieving an SVR in 30% with histologic improvement [95]. These studies were, however, limited by small sample sizes and a lack of randomized controlled trials. 

Carri*ó*n et al. [96] studied patients with mild HCV recurrence (fibrosis stage F0-2) that were randomized into two groups; group A, no treatment; group B, treated with combined peg-IFN-alpha-2b (1.5 *μ*g/kg/week) and RBV (adjusted for renal function, maximum dose 1200 mg/day) for 48 weeks. Median time to treatment was 14 months. An SVR was achieved in 0 (0%) and 13 (48%), respectively. Histologic stabilization or improvement, which corresponded with improvement in the hepatic venous pressure gradient, was recognized with an SVR. Early virologic response was an independent significant factor predicting SVR in the study, as described previously [97–99]. Dose reduction was necessary in 67% of cases, and interruption was necessary in 56%. An increased risk of rejection in the treatment groups was not substantiated. The study demonstrated the advantage of antiviral treatment with peg-IFN and RBV, which achieves permanent viral clearance in a high proportion of patients when initiated at the stage of mild HCV recurrence.

One systematic review [100] of the efficacy of post-transplant treatment with standard IFN (IFN-RBV) [93, 101–117] or peg-IFN in combination with RBV (peg-RBV) [77–80, 118–120] given for 6 to 12 months included 38 studies. Patients were predominantly men with a high rate of genotype 1 infection (>80%). IFN-RBV was associated with an end-of-treatment virologic response rate of 34% and an SVR rate of 24%, while peg-RBV was associated with an end-of-treatment virologic response rate of 42% and an SVR rate of 27%. Pooled discontinuation rates were 24% with IFN-RBV and 26% with peg-RBV. Only 33% with IFN-RBV and 21% with peg-RBV completed the intended protocol. The majority of patients required dose reduction. The overall rate of acute graft rejection was 2% with IFN-RBV and 5% with peg-RBV. The authors concluded that combination therapies have similar tolerability and safety, but the advantage of peg-RBV in terms of viral response remains unclear, and further studies are required.

Another systematic review [121] focused only on studies using peg-IFN in combination with RBV as post-transplant treatment. The 19 reviewed studies showed a mean end-of-treatment response of 42% and a mean overall SVR of 30%. A recent study [122] disclosed an SVR rate of 64% in DDLT patients. The antiviral treatment regimen comprised pegylated-IFN (180 *μ*g) every 2 weeks and RBV at a dose of 200 to 400 mg every day. The treatment duration was flexible and individualized, and it depended on the viral response to treatment. The dosage of tacrolimus was decreased gradually.

## 8. Nonresponders

When an SVR cannot be achieved, the next possible best step is to prevent the progression of fibrosis. Peg-IFN may be histologically beneficial even when HCV eradication is not obtained [123, 124]. Studies of nonresponders to determine the optimal dosage and duration of either IFN, RBV, or both are lacking in terms of delayed disease progression. Administration of oral ursodeoxycholic acid may be beneficial in terms of improving the biochemical response, but not histologic changes, as recently demonstrated in a large-scale, multicenter, double-blind trial in a nontransplant population [125]. It has no clear benefits in liver transplant recipients [126]. 

In a subset of patients with a progressive cholestatic variant of HCV disease [127] despite combined IFN and RBV therapy, indefinite continuation of antiviral therapy [128], or temporary treatment with double-filtration plasmapheresis [129, 130], a novel option to decrease HCV viral load, in combination with antiviral therapy may be effective [130]. Further studies focusing on the long-term risks and benefits of various treatment modalities for patients nonresponsive to antiviral therapy are necessary.

## 9. Future Perspectives

Previous descriptions [131] as well as the descriptions above indicate the need for additional treatment modalities. The treatment goal should be eventual viral eradication. In theory, this goal can be achieved by disrupting the steady-state HCV kinetics by reducing virion production, thereby allowing infected cells to be eliminated [132, 133]. Lang [134] classifies the future drugs currently under investigation into four categories: new IFNs, RBV alternatives, specific HCV inhibitors, and immunomodulators. Some of these drugs are in phase III trials awaiting further clinical evaluation.

As tolerability is the primary problem of the current widely applied combined IFN and RBV treatment after liver transplantation, specific HCV life-cycle inhibitors may have a beneficial role. Among the various drugs under investigation, protease inhibitors such as telaprevir and boceprevir appear to be safe and are currently undergoing clinical evaluation. A clinical trial with triple combinations of peg-IFN, RBV, and telaprevir has been performed [135] in patients with chronic HCV. The SVRs of the patients administered telaprevir for 24 and 48 weeks were 61% and 67%, respectively. High SVR was obtained in patients who had not had a sustained response to the therapy with peg-IFN and RBV [136]. The SVR of the control patients (peg-IFN and RBV for 48 weeks) was 41%. A phase-III clinical trial of boceprevir was begun in 2008 [137–140]. None of the new anti-HCV drugs, however, are currently being evaluated in HCV-infected liver transplant recipients.

## 10. Conclusions

HCV continues to be a major challenge in liver transplantation. Reinfection is common after transplantation, and progression of the disease leads to a dismal outcome. Pretransplant antiviral therapy with at least on-treatment virologic response at the time of transplantation would be desirable, but the tolerability and risk of therapy limits the applicability to patients with compensated or mildly decompensated liver disease (low MELD score). Preemptive treatment is also limited by frequent complications in the early post-transplant phase and a high rate of side effects. The use of combined peg-IFN and RBV therapy has increased the rate of viral clearance, but its intolerability in the majority of patients prevents its general application. The overall risk and benefit of the current strategy over the long term remains to be evaluated. Additional modalities combining new drugs, such as protease inhibitors, should be pursued.

## Abbreviations

DDLT: deceased donor liver transplantation
HCC: hepatocellular carcinoma
HCV: hepatitis C virus
INF: interferon
LDLT: living donor liver transplantation
MELD: model for end stage liver disease
Peg-IFN: pegylated interferon
SVR: sustained viral response
RBV: ribavirin.
